# Profile of professionals working in intradialytic exercise programs in Brazil: a national survey

**DOI:** 10.1590/2175-8239-JBN-2021-0264

**Published:** 2022-02-28

**Authors:** Fabrício Sciammarella Barros, Bruno Valle Pinheiro, Heitor Siqueira Ribeiro, Francini Porcher Andrade, Camila Rodrigues de Souza, Amanda Cruz do Nascimento Amorim, Leda Marília Fonseca Lucinda, Maycon Moura Reboredo

**Affiliations:** 1 Universidade Federal de Juiz de Fora, Núcleo de Pesquisa em Pneumologia e Terapia Intensiva, Juiz de Fora, MG, Brasil.; 2 Universidade Federal de Juiz de Fora, Faculdade de Medicina, Juiz de Fora, MG, Brasil.; 3 Universidade de Brasília, Faculdade de Educação Física, Brasília, DF, Brasil.; 4 Universidade da Maia, Centro de Investigação em Desporto, Saúde e Desenvolvimento Humano, Porto, Portugal.; 5 Universidade Federal do Rio Grande do Sul, Programa de Pós-Graduação em Ciências Pneumológicas, Porto Alegre, RS, Brasil.

**Keywords:** Renal Dialysis, Exercise, Rehabilitation, Diálise Renal, Exercício físico, Reabilitação

## Abstract

**Objective:**

This survey was designed to assess the profile of professionals working in intradialytic exercise programs (IEPs) in Brazil and reveal the motivators and barriers they face.

**Methods:**

The survey was sent to physiotherapists and exercise physiologists working in IEPs in Brazil. Phone interviews and electronic forms were used to collect the answers to the survey questionnaire.

**Results:**

Forty-one of the 261 included dialysis centers had IEPs; 44 professionals answered the questionnaire over the phone and 26 used the electronic form to do it. A total of 70 professionals (mean age 33.4±7.4 years; 84.3% physiotherapists) answered the questionnaire. Resistance training was the preferred mode of therapy. Most of the IEPs were connected to research and were paid for by private health insurance. The desire to work in a different field (30.0%) and lack of resources (31.4%) were the most prevalent motivator and barrier cited by IEP professionals working in dialysis centers, respectively.

**Conclusion:**

The majority of the few professionals that work in IEPs in Brazil are physiotherapists. Lack of resources was the most commonly reported barrier faced by survey respondents.

## Resumo

**Objetivo:**

Avaliar o perfil dos profissionais que atuam em programas de exercício físico intradialítico (EFI) no Brasil e as motivações e barreiras para a atuação desses profissionais.

**Métodos:**

Foram incluídos fisioterapeutas e profissionais de educação física que atuam em programas de EFI no Brasil. Para coletar os dados, foi aplicado um questionário por ligação telefônica ou formulário eletrônico.

**Resultados:**

Dos 261 centros de diálise analisados, 41 apresentaram programas de EFI, sendo que 44 profissionais responderam ao questionário por telefone e outros 26, por meio eletrônico. Foram avaliados 70 profissionais (33,4±7,4 anos, 84,3% fisioterapeutas). A modalidade terapêutica mais aplicada foi o treinamento resistido. A maioria dos programas de EFI está associada a atividades de pesquisa e é financiada pela saúde suplementar. A possibilidade de atuação em um novo campo de trabalho (30,0%) e a falta de recursos (31,4%) foram a motivação e a barreira mais prevalentes para atuação dos profissionais nos centros de diálise, respectivamente.

**Conclusão:**

Poucos profissionais atuam em programas de EFI no Brasil, sendo a maioria fisioterapeutas. A falta de recursos foi a barreira mais prevalente para a atuação desses profissionais.

## Introduction

intradialytic exercise training (IET) has been recommended to patients with chronic kidney disease on hemodialysis^1-4^. Previous studies found that IET leads to lower blood pressure levels and improvements in functional capacity, anemia, muscle strength, muscle oxidative metabolism, and quality of life^2,3,5^. Additionally, recent meta-analyses confirmed that IET is safe and provides additional benefits such as improved dialysis effectiveness and positive impacts on patient mood^4,6,7^.

However, the implementation of intradialytic exercise programs (IEPs) is not an obstacle-free endeavor. Our national survey found that only 41 dialysis centers in Brazil offer IET to their patients, with lack of resources ranking atop the list of barriers faced by centers offering IEPs and centers that have tried and failed to implement IEPs^8^. Ma et al. also found that lack of resources was the main barrier to the establishment of IEPs in dialysis centers in Ontario, Canada^9^.

Although lack of resources has been recognized as a barrier to the implementation of IEPs, little is known about the professionals working in these programs. Learning more about them might help develop strategies to foster the implementation of IEPs.

Therefore, this study was designed to look into the profile of professionals working in IEPs and assess the motivators and barriers they face while working in dialysis centers in Brazil.

## Methods

### Population

This cross-sectional study was conducted from April 2019 to June 2020. It was part of a large survey involving dialysis centers in Brazil^8^. Consent from study participants was given over the phone or via the electronic form. The Ethics Committee of the Federal University of Juiz de Fora approved the study protocol (nº 3.054.613).

The population included in the study comprised physiotherapists and exercise physiologists working in IEPs in Brazil. Participants who failed to send their answers after they were contacted five times and individuals sending incomplete forms in were excluded.

### Study protocol

On April 1, 2019, the search for participants was initiated from a list of dialysis centers registered with the Brazilian Society of Nephrology. Clinics not offering ambulatory hemodialysis and centers with registration inconsistencies were excluded. Eligible centers were sent an e-mail inviting them to join the study. At a later moment, they were contacted by phone in a standard scripted call. The physiotherapists and exercise physiologists working with IET at the included centers were interviewed over the phone using a standard questionnaire as reference for data collection. An active search for professionals working in dialysis clinics in Brazil was also performed based on the attendance records of a meeting about IET. These individuals were contacted by e-mail and were asked to answer a questionnaire on Google Forms.

### Assessment

The research study authors developed the questionnaire based on prior studies looking into the profile of professionals working with physical rehabilitation^9,10^. With the specific profile of the professionals included in this study in mind, the questionnaire was adjusted to contemplate questions about IET.

The following data were captured from participants: age, sex, education (attendance to undergraduate, specialization, and/or graduate programs; type of educational institution; curses in the field of nephrology), type of engagement with the dialysis center, time working in this area, and number of scientific papers read monthly. Business organization information from the included dialysis centers and data about the types of sessions held with patients and the IEPs in place were collected. The motivators and barriers affecting professionals working in dialysis centers were also assessed.

### Statistical analysis

Results were expressed as mean values ± standard deviation, medians (interquartile range) or proportions, as required. Descriptive statistics was used in the assessment of data from professionals, centers, IEPs, motivators, and barriers. The professional-to-patient ratio was calculated based on the number of professionals in each State and the estimated total number of patients per State as described in the Dialysis Census of the Brazilian Society of Nephrology. Statistical analysis was performed with the aid of software program SPSS 17.0 for Windows (SPSS Inc., Chicago, USA).

## Results

**Figure 1:**
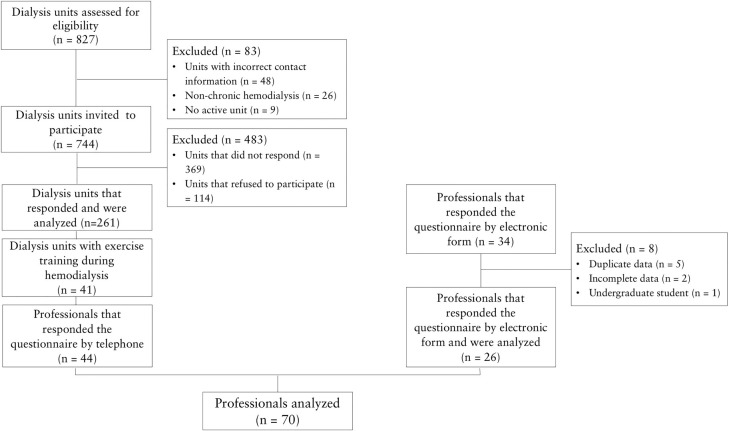
Flowchart showing analyzed professionals.

Eighty-three of the 827 dialysis centers evaluated for eligibility were excluded and 744 were initially included in the study. A total of 369 centers were later excluded, 114 refused to join, 261 were reviewed, 41 had IEPs, and 44 professionals answered the questionnaire over the phone. Another 34 professionals answered the electronic form questionnaire, of which eight were excluded and 26 included in the study (Figure 1).

**Figure 2:**
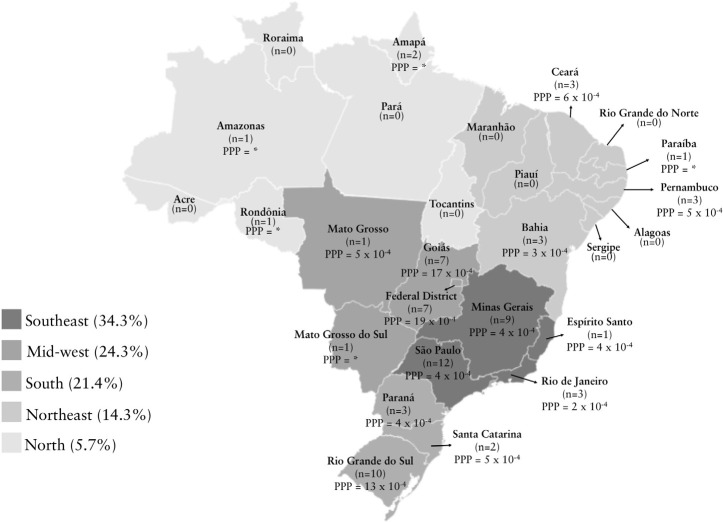
Number of professionals working in intradialytic exercise programs in each Brazilian State.

Note: PPR = professional-to-patient ratio. * ratio not calculated due to missing data in the Brazilian Dialysis Census.

A total of 70 professionals working in IEPs were included. The vast majority included (84.3%) physiotherapists; they were aged 33.4±7.4 years on average; individuals of the female sex prevailed (Table 1). The distribution of professionals per Brazilian State and the professional-to-patient ratio are illustrated in Figure 2.

**Table 1:** Characteristics of professionals, dialysis units, intradialytic exercise programs, and reported motivators and barriers to working in ieps

Variables	N = 70
*Professionals*	
Women, n (%)	49 (70)
Age, mean ± SD, years	33.4 ± 7.4
Physiotherapists, n (%)	59 (84.3)
Exercise physiologists, n (%)	11 (15.7)
Time on the job since graduation, mean ± SD, years	9.1 ± 7.6
Type of university, n (%)	
Private	56 (80)
Public	14 (20)
Specialization course, n (%)	21 (30)
Master's degree, n (%)	14 (20)
Doctoral degree, n (%)	8 (11.4)
Course in nephrology, n (%)	7 (10)
Number of scientific articles read per month, mean ± SD	6.2 ± 5.2
Years working in nephrology, median (IQ)	2.0 (4.6)
Type of work performed, n (%)	
Research	25 (35.7)
Contractor	22 (31.4)
Employee	12 (17.1)
Civil servant	7 (10)
Informal worker	4 (5.7)
*Characteristics of dialysis units and intradialytic exercise programs*	
Private center, n (%)	56 (80)
Public center, n (%)	14 (20)
Funding, n (%)	
Brazilian Public Healthcare System	14 (20)
Private health insurance	26 (37.1)
Either	29 (41.4)
Exercise components*, n (%)	
Resistance exercises	62 (88.6)
Aerobic exercises	49 (70)
Stretching exercises and manual therapy	49 (70)
Breathing exercises	44 (62.9)
Combined training**	19 (27.1)
Neuromuscular electrical stimulation	10 (14.3)
*Motivators to work in dialysis units, n (%)*	
Desire to work in a different field	21 (30)
Research	20 (28.6)
Patient demands and needs	16 (22.9)
Interest in the area	6 (8.6)
Career opportunity	5 (7.1)
Influence from colleagues, teachers and students	4 (5.7)
Invitation to work in this area	4 (5.7)
Job innovation and salary	2 (2.9)
*Barriers to work with intradialytic exercise programs in dialysis units, n (%)*	
Not reported	25 (35.7)
Lack of resources	22 (31.4)
Lack of human resources	15 (21.4)
Staff resistance	14 (20)
Poor patient compliance to exercise training	13 (18.6)
Unit management resistance	10 (14.3)
Lack of research protocols	2 (2.9)
Low salary	1 (1.4)

* Note that the sum is greater than 100% because a professional could respond more than one exercise component.

** Aerobic and resistance training.

The most commonly used mode of therapy was resistance training. The desire to work in a different field and lack of resources were the most prevalent motivator and barrier cited by IEP professionals working in dialysis centers, respectively (Table 1).

## Discussion

This study looked into the profile of professionals working in IEPs in Brazil. The main findings for the 70 professionals included in the study were: most of them were physiotherapists involved in research; the most commonly used mode of therapy was resistance training; the desire to work in a different field was the main motivator behind the choice to work at a dialysis center; and lack of resources stood out as the main barrier faced by professionals working in this field.

Few professionals work in IEPs in Brazil and the professional-to-patient ratio in the nation is quite low, which serves as evidence of the fact that few patients on hemodialysis are offered IET. The most recent Brazilian dialysis census indicated that an estimated 136,691 individuals were on dialysis in Brazil. Our study found that only 5,360 patients were on IEPs^8,11^.Another relevant finding was the biased geographic distribution of the 70 included professionals, who were simply inexistent in nine Brazilian States. The absence of a legal requirement to make physical rehabilitation professionals available in dialysis centers might serve as an explanation for the incredibly low number of such professionals in dialysis centers^12^. Lack of professionals ranked second in the list of barriers to working in dialysis centers. Similarly, lack of human resources was the second most mentioned barrier reported by participants of a Canadian study^9^.

Our study showed that most of the professionals working in IEPs were physiotherapists. A Canadian study also found that physiotherapists were involved in IEPs^9^. Interestingly, long term intradialytic exercise programs from four different countries described in the literature also reported the relevant involvement of other professionals, such as nurses^13^. However, the authors found that programs were implemented and managed more adequately when professionals with expertise on the field were involved^13^. This finding was recently confirmed in a study that showed that the presence of a physiotherapist in a dialysis center more than doubled patient compliance to IET^14^.

In Brazil, a significant portion of the professionals working in IEPs at dialysis centers were also involved with research and education. Similarly, Ma et al. found that 38% of the evaluated dialysis centers had ties with teaching and research institutions^9^.The authors of a Portuguese study found that the benefits evinced in a research project about IET led to the introduction of IEPs in 37 dialysis centers in the nation, eventually elevating IET to the status of routine clinical practice^13^.

Resistance training was the most commonly prescribed mode of therapy. Considering the data from clinical trials and meta-analyses about IET, aerobic exercises are preferentially prescribed^1,4,6^. The greater use of resistance training in Brazil is possibly due to the lower cost of exercise equipment and the relative simplicity of training protocols.

Most of the interviewed professionals said that the main reason behind the choice of working at a dialysis center was the desire to work in a different field. Few professionals took courses in nephrology and most had not been on the job for long. Unfortunately, most professional boards still do not recognize the role of rehabilitation in nephrology, a fact reflected in the lack of attention given to hemodialysis patient care in formal training curricula. As seen in other studies, lack of resources was the most prevalent barrier to working with IET in dialysis centers^9,15^.

From a practical point of view and considering that most IEPs are connected to research activity and are paid for by private health insurance, new efforts must be made so that IET is paid for by the Brazilian Public Healthcare System and an effective procedure is offered at more dialysis centers in Brazil.

Our study has its limitations. Although it included centers from many Brazilian States, our findings cannot be generalized or applied to describe the reality faced by every professional working with IET in the nation. The costs and funding available for IEPs were not assessed, despite their value in aiding in the interpretation of our results.

We found that few professionals work in IEPs in Brazil. Most are physiotherapists involved with research. We also found that the most common motivator and barrier to working at a dialysis center were the desire to work in a different field and lack of resources, respectively.
